# Impact of Anxiety on Readiness for COVID-19 Vaccination among Polish Nursing Undergraduate Students: Nationwide Cross-Sectional Study

**DOI:** 10.3390/vaccines9121385

**Published:** 2021-11-24

**Authors:** Joanna Gotlib, Mariusz Jaworski, Dominik Wawrzuta, Tomasz Sobierajski, Mariusz Panczyk

**Affiliations:** 1 Department of Education and Health Sciences Research, Faculty of Health Sciences, Medical University of Warsaw, 02-091 Warsaw, Poland; joanna.gotlib@wum.edu.pl (J.G.); mariusz.jaworski@wum.edu.pl (M.J.); mariusz.panczyk@wum.edu.pl (M.P.); 2 Faculty of Applied Social Sciences and Resocialization, University of Warsaw, 00-325 Warsaw, Poland; tomasz.sobierajski@uw.edu.pl

**Keywords:** undergraduate nursing students, COVID-19 vaccine, knowledge, anxiety, GAD-7

## Abstract

The COVID-19 pandemic had a huge impact on the mental health of people around the world, and it increased the level of fear of infection and anxiety about the consequences of the disease caused by the SARS-CoV-2 virus. We examined the relationship between the level of anxiety among nursing students and their knowledge about COVID-19 vaccination. In addition, we explored the correlations among the level of anxiety, knowledge about vaccination, and the willingness to vaccinate against COVID-19. A total of 790 undergraduate nursing students participated in the study. The results demonstrated that the level of anxiety among the surveyed nursing students was low; 40% of the study participants did not report any anxiety at all, 30% reported mild anxiety, 20% reported moderate anxiety, and 9% reported severe anxiety. At the time of the study, 77.2% of the participants were already vaccinated against COVID-19. Student knowledge about vaccination against COVID-19 was high and anxiety levels were low, with no direct correlation between the knowledge of vaccination and the severity of anxiety.

## 1. Background

### 1.1. Introduction

The COVID-19 pandemic seriously impacted various aspects of society on a global scale [1] and placed severe psychological stress on people around the world [2]. During the COVID-19 pandemic, concerns about mental health and substance abuse grew, including concerns about suicidal ideation [3]. In January 2021, 41% of adults reported symptoms of anxiety and/or depressive disorders, a share that was largely stable since spring 2020 [4]. It is believed that high levels of stress and anxiety related to the fear of infection, as well as uncertainty about one’s future, are among the most frequently observed symptoms [5,6,7,8].

The COVID-19 pandemic also initiated a period of new and difficult challenges for nursing students. Increased anxiety among nursing students during the COVID-19 pandemic was often discussed, mostly in the early stage of the pandemic, usually analyzed in relation to student knowledge about the SARS-CoV-2 virus [9,10,11]. The occurrence of increased anxiety has also been addressed in many publications in the context of changes in education during the pandemic, the moving of most classes to online [12,13,14,15], the fear of senior year students about graduation [16], and the impact of the pandemic on the professional identity of students [17]. The influence of increased anxiety on the occurrence of abnormal health behaviors and various types of disorders among students, such as eating or sleep disorders [18,19,20], also turned out to be an important problem, significantly reducing the quality of student life [9,10,11].

The research results presented in the vast majority of publications have confirmed the existence of a relationship between student knowledge about the SARS-CoV-2 virus and anxiety levels. The greater the knowledge, the lower the anxiety and the more frequent the compliance with recommendations and restrictions related to the pandemic [16]. In the initial period of the pandemic, due to the lack of sufficient research, open access to evidence-based knowledge about the new type of coronavirus was limited. Now, a year into the pandemic, knowledge about COVID-19 significantly increased [21].

The development of a safe vaccine against COVID-19 is the most important discovery, not only from a scientific but, above all, from a social point of view [22]. The vaccination program currently being rolled out around the world comprises the most important and effective step toward ending the SARS-CoV-2 pandemic. In many countries in which the majority of citizens were vaccinated (e.g., the United Kingdom, the United States, and Israel), the pandemic situation is clearly stabilizing, which is indicated by the decreasing number of new infections and deaths due to COVID-19 [23,24,25].

The above-mentioned factors, that is, open access to reliable knowledge about the COVID-19 pandemic, access to vaccination, and the possibility of being vaccinated, should contribute to a gradual decrease in anxiety levels. On the contrary, anxiety and stress have a negative impact on the learning process, the acquisition of knowledge, and selective attention [26]. This can be a crucial negative factor hindering learning about COVID-19. This may be especially important in the context of nursing students who need to acquire new knowledge in difficult and often stressful conditions. Therefore, information on COVID-19 and vaccination should not only be reliable, but also properly communicated.

To the best of our knowledge, none of the available publications have analyzed anxiety levels in nursing students in relation to their knowledge about vaccination against COVID-19, as well as the possibility and willingness to take the vaccine.

The authors of the publication assumed that the lower the level of knowledge of students about vaccination against COVID-19, the higher the level of anxiety in the studied group of students. Moreover, the higher the level of knowledge about the COVID-19 vaccination, the greater the willingness to accept the vaccine and the lower the level of anxiety.

### 1.2. Aim of the Study

To determine the relationships among the anxiety levels, knowledge levels about COVID-19 vaccines, and willingness of undergraduate nursing students to be vaccinated.

## 2. Materials and Methods

### 2.1. Design and Setting

A cross-sectional, national online survey study was conducted from March to April 2021. A total of 12 universities that offer undergraduate nursing programs in Poland were invited to participate in the study.

### 2.2. Local Context

The participation of nursing students in the nationwide vaccination campaign was voluntary. Those willing to vaccinate could book a vaccination appointment via the online system. After logging into an individual account, they could choose a vaccination time and date from a list of available vaccination sites. The appointment was confirmed by e-mail. One day prior to their vaccination appointment, a reminder was sent by the system. It was also possible to cancel the vaccination appointment or change the previously booked time, date, or vaccination site.

### 2.3. Participants and Sample Size

Students enrolled in a three-year-long undergraduate nursing study program were eligible for this study. The total potential number of study participants was 470,000 students from the 12 universities. The participants were recruited by coordinators who were employees of the 12 universities that had agreed to participate in the nationwide study. Each coordinator was knowledgeable about the purpose of the study, the distribution method of the research tool, and the principles of supervision over data collection. In total, 850 students declared their participation in the study. However, the data package was returned by 793 students and 790 questionnaires were selected for final analysis. The 3 remaining were incomplete. The sample was representative of a broader spectrum of Polish nursing students. With this sample size and the total number of nursing students in Poland (*N* = 7000), the error margin was 2.00% (95% confidence level and a proportion of 0.75).

### 2.4. Instruments

The questionnaire used in this study was an original questionnaire created for the purpose of this study. Survey development was informed by the extant literature on COVID-19, vaccines against COVID-19, and vaccine readiness and hesitation among medical staff and medical and nursing students. The survey was also based on our previous research experience [27]. The questionnaire consisted of the following three sections: (1) demographics; (2) motivations and attitudes toward COVID-19 vaccines; (3) vaccine information sources. The questionnaire used Likert-scale, closed-ended, semiopen, and open-ended questions. It took approximately 25 min to complete the online questionnaire.

A total of 18 items in the survey included the following demographics: year and level of the program, sex, age, place of residence, chronic illnesses, flu vaccine uptake, career plans, the undertaking of student internships in departments treating COVID-19 patients, information about contracting COVID-19 (for the study participant and/or their immediate family), the course of the disease, information on receiving the vaccine, vaccination site, and the occurrence of adverse postvaccination reactions.

The questions about motivation, firstly, regarded information about the extent to which caring for oneself and for loved ones contributed to the decision to vaccinate. A total of four items in the survey included questions regarding attitudes toward COVID-19 vaccines. The last section of the questionnaire included questions about the frequency of using vaccine information sources (13 items in total).

The questionnaire also assessed anxiety levels. Anxiety levels were assessed using the Generalized Anxiety Disorder 7-Item Scale (GAD-7) [28,29,30], which is a seven-item scale based on a four-point Likert scale that is used to assess anxiety levels, as well as the risk of a generalized anxiety disorder (GAD). The questions included in the survey enabled the respondents to assess the intensity of anxiety, tension, nervousness, the ability to control these feelings, the ease with which they occur, and problems with relaxation. In each question, 0–3 points could be awarded depending on the frequency of the occurrence of the phenomenon in the last 14 days (0—not at all; 1—every few days; 2—more frequently than half the days; 3—almost every day). A result of 5, 10, or 15 points indicated the presence of mild, moderate, or severe anxiety, respectively. Obtaining at least 10 points indicated a high probability of the occurrence of a generalized anxiety disorder [28]. The GAD-7 was used with a suggested cut-off level of 10 points for defining moderate anxiety, and a cut-off level of 15 points for defining severe anxiety (GAD-7 has the maximum point of 21). This questionnaire was widely used and is reported to have high internal consistency and good test–retest reliability among adults [31,32], adolescents [28], and college students [33].

The survey was piloted with 20 nursing faculty members and 20 nursing students using Delphi study, which means that the selected group of students who participated in the pilot study after completing the questionnaire were asked about specific issues in the questionnaire to check whether the questionnaire was logically, methodologically, conceptually, and linguistically correct. Our survey was prepared according to Delphi Survey Technique guidelines [34]. Data collection comprised three rounds. In the first Delphi round, the members of the expert panel received a questionnaire containing thirty open-ended questions. Their answers from the first round were summarized in mind maps. For each participant’s background (nursing faculty members and nursing students) a separate mind map was created. In the second round, the participants received a follow-up questionnaire containing twenty-four questions based on the summary from the first round. Answers from the second round were summarized in mind maps as well. In the third round, a face-to-face discussion meeting was organized among the experts: nursing faculty members and nursing students, based on mind maps and the questionnaire. The aim of the discussion was to validate the findings from the first two rounds by reaching a consensus on the final version of the questionnaire. During this final stage, the panel of experts decided to delete six questions, as they repeated similar issues. Revisions were made to improve the clarity of the statements. The survey is also available in English upon request.

### 2.5. Data Collection

The questionnaire was distributed using the LimeSurvey web platform. The link to the survey was made available to 11 coordinators at the participating universities. The type of survey distribution used resulted from the limited possibility of direct contact with the respondents due to the restrictions introduced by the Minister of Health in connection with the COVID-19 pandemic. In such a situation, online research is a recommended approach that allows one to quickly reach the study group and ensure its safety [35,36].

### 2.6. Ethical Considerations

The study protocol was approved by the ethics committee of Medical University of Warsaw (IRB approval no. KB/76/2021). Before the study, the participants were informed about the principles of anonymity and confidentiality during data collection. No personal data, including computer IP addresses, were collected. Based on the data collected, analyzed statistically, and presented below, it is impossible to identify the survey participants.

### 2.7. Data Analysis

Quantitative and categorical variables are described with descriptive statistical methods. For quantitative variables, the following measures were determined: central tendency (mean, M) and dispersion (standard deviation, SD). For the categorical variables, the following measures were determined: number (*N*) and frequency (%).

Cross-tables and a chi-squared test were used to assess the impact of the level of perceived anxiety on the selected categorical variables. To assess the impact of the level of perceived anxiety on the selected quantitative variables, one-way ANOVA was used.

All calculations were performed with STATISTICATM 13.3 software (TIBCO Software, Palo Alto, CA, USA). For all analyses, a *p*-level of <0.05 was considered statistically significant.

## 3. Results

### 3.1. Sample Characteristics

A total of 790 Polish undergraduate nursing students participated in the study. The largest group of study participants consisted of second-year students (*N* = 331, 41.9%) and women (*N* = 721, 91.3%), which is similar to the average gender distribution in nursing faculties in Poland. The mean age of the study participants was 22.4 (SD = 5.00). The sample group was ethnically homogeneous. The selected characteristics of the study group are presented in Table 1.

### 3.2. Anxiety Level

**Figure 1:**
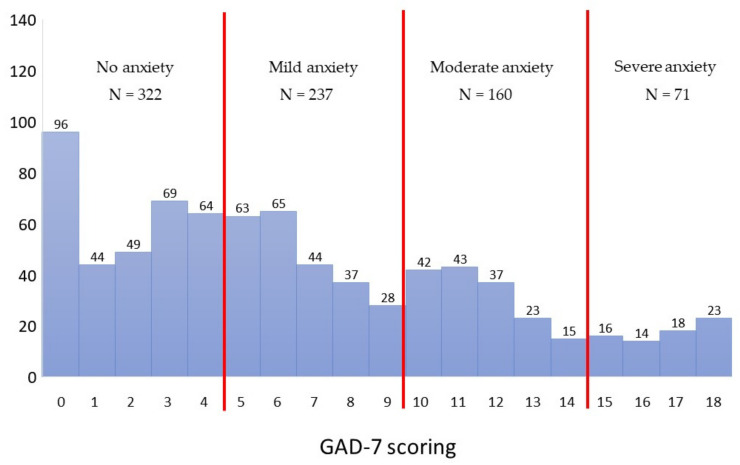
Distribution of GAD-7 scoring (*N* = 790).

The mean score obtained on the GAD-7 scale was 6.58 (SD = 5.01), with a minimum value of 0.0 and a maximum value of 18.0. The score obtained from the GAD-7 measurement was characterized by a moderate right-sided skewness (skew = 0.56), which indicates that there was a slightly higher number of people with lower scores. The measurement of the level of anxiety showed that 40.8% of the study participants (*N* = 322) did not report any anxiety at all, another 30.0% (*N* = 237) reported mild anxiety, 20.2% (*N* = 160) reported moderate anxiety, and 9.0% (*N* = 71) reported severe anxiety (Figure 1).

### 3.3. Anxiety Level and Willingness to Get a COVID-19 Vaccine

The vast majority of the study participants were already vaccinated against COVID-19 at the time of the study (*N* = 610, 77.2%), most often with an mRNA vaccine (*N* = 482, 61.0%) at their university’s vaccination site (*N* = 503, 63.7%). The analysis of the potential influence of the anxiety level on the decision to vaccinate did not show a statistically significant correlation in this regard. In addition, the choice of vaccination site was not dependent on the measured anxiety level (Table 2).

### 3.4. Anxiety Level and Vaccine Information Sources

Among the surveyed students, 40.3% (*N* = 318) of the study participants indicated that the university provided access to up-to-date knowledge on vaccination. There was no statistically significant difference in the level of perceived anxiety regarding access to information on vaccination depending on the university (Table 3).

Among the various sources of information from which the respondents obtained knowledge on vaccination, five showed a different frequency of vaccination depending on the perceived level of anxiety (Table 4). The respondents from the “no anxiety” group, on average, least frequently used such sources of information on vaccination as specialist professional publications, social media, blogs and vlogs of specialists, nonspecialist blogs and vlogs, and information obtained from other students at university.

### 3.5. Anxiety Level and Knowledge about Vaccination and Vaccines

The analysis of the impact of the level of perceived anxiety and knowledge about selected aspects of COVID-19 vaccination showed no statistically significant relationships (Table 5). In addition, no significant correlation was found between the level of anxiety of the surveyed students and the knowledge about contraindications to vaccination (Table 6).

## 4. Discussion

### 4.1. Findings

The anxiety levels in the surveyed group of nursing students did not differ significantly from the anxiety levels of the population of medical and nonhealth science students before the COVID-19 pandemic. The largest group of respondents (40%) did not report any anxiety at all, 30% reported mild anxiety, 20% reported moderate anxiety, and only 9% reported severe anxiety. Similar results of studies on the occurrence of anxiety among students before the pandemic were obtained from university and college students in Hong Kong (12.2% had moderate anxiety, and 5.8% had severe anxiety) [37], Portugal (15.6% had moderate anxiety, and 8.3% had severe anxiety) [33], and Australia (17.5% had moderate anxiety) [38]. Among medical students, the prevalence of moderate anxiety was 25% in the United Kingdom, 20% in North America, 13.7% in New Zealand, and 23% in Lebanon [39].

However, the available analyses of anxiety levels conducted among students at the beginning of the COVID-19 pandemic differ significantly from the results of our study; the level of anxiety among students was significantly higher at that time—the vast majority of American students (84%) declared feeling anxious or overwhelmed [13], and as many as 13.1% of students from Israel reported severe anxiety [19]. The discrepancy in the results can be explained, among other things, by the ability to quickly adapt to new conditions. Such adaptation allows one to quickly restore mental balance and, thus, reduce emotional tension [13,19].

The main factors contributing to the occurrence of high levels of anxiety at the onset of the COVID-19 pandemic were primarily related to the emergence of a new type of coronavirus. The high level of anxiety in the group of students resulted mainly from the lack of knowledge about the new type of coronavirus, its routes of transmission, and the methods of COVID-19 diagnosis and treatment. This possibly had a negative impact on the mental health of students (e.g., increased stress and anxiety) and possibly modified the cognitive abilities of students, for example, by suspending the selectivity of attention [26]. The fear of infection and the more common fear of infecting loved ones were important factors triggering anxiety. Issues directly related to studying during the pandemic were also significant factors causing anxiety among students, including the shift to online education, the limited possibility of clinical practice experiences, and developing the most important practical nursing skills. On the contrary, after the resumption of clinical practice experience at the patient’s bedside in a modified form, the students were also afraid of attending practical classes in wards treating COVID-19 patients. Anxiety resulted from the fear of infection caused by a lack of an appropriate amount of personal protective equipment and the inability to use it properly [10,19].

Many publications also emphasized fear about graduating as planned and the possibility of entering the job market as important factors affecting the occurrence of increased levels of anxiety among nursing students [10]. This situation mostly concerned students who were scheduled to graduate in the 2019/2020 academic year [16,17,18,19]. Moreover, the high level of anxiety among nursing students at the beginning of the pandemic also negatively affected their professional identification and career goals [20,21].

Another factor that possibly affected the increased level of anxiety could were economic, that is, the inability to take up temporary work by students (e.g., in tourism or gastronomy) or to obtain additional sources of income [18,19,20,21]. Branches of those industries in which students are most frequently employed part-time (such as tourism and gastronomy) were most severely impacted economically by the pandemic; therefore, this economic factor could have played a crucial role in increasing anxiety levels among students, particularly at the onset of the pandemic [17,18,19,20].

To the best of our knowledge, the research results presented in this paper are the first to describe anxiety levels among nursing students after more than a year since the onset of the COVID-19 pandemic, at a time when it is possible to receive the COVID-19 vaccine, and when most medical students in Poland were already vaccinated [40].

The COVID-19 National Vaccination Program in Poland assumed that the distribution of vaccines in society would be gradual and that the vaccination of society would be carried out in stages. Based on the demographic and occupational characteristics of Polish society, several social groups were selected to receive COVID-19 vaccines in a pre-defined order and stages. The vaccination program was divided into four stages, from Stage 0, comprising those to receive the vaccination first, to Stage III, at which point all willing adults to be vaccinated. The risk of infection, risk of developing a serious illness or death, and the risk of disease transmission were taken into account when determining the target groups for each stage.

Stage 0, the priority stage, which began vaccination on 27 December 2020, comprised employees of the healthcare sector, administrative and support staff of care homes and medical institutions, and academic teachers and students of medical faculties. Moreover, medical schools organized vaccinations for their students at university sites, which certainly made it easier for students to get vaccinated. For this reason, at the time of the study (March/April 2021), the vast majority of respondents (77.2%), who were nursing students from medical universities all over Poland, were vaccinated against COVID-19.

According to the authors, several factors contributed to the low level of anxiety in the studied group of Polish nursing students, the most important of which were getting vaccinated as soon as possible and having high levels of knowledge about COVID-19 vaccination. In the presented research, the level of knowledge about vaccination was high, while the level of anxiety was low. At the same time, the level of student knowledge about vaccination against COVID-19 did not significantly affect the level of anxiety. These results are consistent with the results of other studies [17,18,19,20,21]. In the majority of the available publications, the level of student knowledge about the pandemic did not significantly affect the level of anxiety. The lack of a direct link between the amount of knowledge and the severity of anxiety may be explained by the fact that students’ concerns are more self-centered (e.g., students are concerned about the end of the academic year and exams). In this context, knowledge about the virus does not directly influence the student’s concern; the impact is indirect.

The vast majority of the study participants gave correct answers to questions about COVID-19 vaccination, and most of them pointed to their home university as the source of their knowledge about said vaccination. These results can be explained by referring to research in the field of the psychology of stress. Those nursing students who experienced severe stress and fear related to the possibility of infection used their personality resources (e.g., cognitive functions) to obtain reliable knowledge about the virus. Their attention could were more selective toward proven actions that would allow them to protect themselves and their loved ones from becoming infected. It was a way of adapting to a new situation. The impact of stress on the learning process depends on, among other things, the physiological changes that take place in the human body, as well as emotions. Emotional events are remembered better, and low levels of stress can strengthen memory. If the material to be learned is related to stress, it will be remembered better [26].

Access to up-to-date knowledge on COVID-19 vaccination, as well as the compositions, types, effects on the body, and effectiveness based on clinical trials and the first months of vaccination, may also have had a great influence on reducing anxiety. Students of medical universities may expect to obtain knowledge about COVID-19 vaccination at university. However, only a minority of 40.3% of students declared gaining such knowledge. The study participants assessed differently whether the university provided them with access to up-to-date knowledge about COVID-19 vaccination, depending on the degree of anxiety. The study participants who did not experience anxiety or described it as mild reported that the university provided them with access to up-to-date knowledge more often than those with moderate or severe levels of perceived anxiety. The reported levels were 41% and 32.4% for students with no anxiety and severe anxiety, respectively. The higher the perceived anxiety, the greater the confidence that the university provided or did not provide up-to-date information on vaccination (25.5% of the students in the no anxiety group and 18.3% in the severe anxiety group declared “I don’t know”).

The distribution of responses in particular groups of perceived anxiety is very interesting, and the answers mainly refer to social media and web portals. It turns out that these two sources: social media and web portals, indicated as potentially causing the greatest anxiety [18,19,20,21] were almost as frequently used as a source of information by all groups, regardless of perceived anxiety. Therefore, it should be assumed that despite the fact that the vast majority of students obtain information on vaccination against COVID-19 from these two sources of information, it did not significantly affect the level of anxiety in the studied group of students.

Another factor that could have significantly reduced the level of anxiety was the fact that the vast majority of the study participants (88%) began their studies during the pandemic. Therefore, the change in the method of education to online education was not much of a change for this group of students. From the very beginning of their studies, most of the students had attended their classes online. Moreover, concerns about completing their studies on time were raised rather by students graduating in June/July 2020. Due to the ongoing pandemic, many universities developed new diploma examination procedures for students graduating in the 2020/2021 academic year. As younger students are now familiar with them, these regulations no longer cause anxiety related to uncertainty. Therefore, it can be said that the quick reactions at the university level improved the mental state of students, especially as far as reducing fear among students is concerned. Quick and effective crisis management was, therefore, essential.

### 4.2. Strengths and Limitations of the Study

In our study, the questionnaire was developed based on the information about COVID-19 available from the WHO and the Polish National Institute of Public Health and Ministry of Health websites. Validation was performed to increase the reliability of our study. Moreover, this study involved a representative group of students from 12 Polish universities, adding value to the research results presented in this paper. Nevertheless, our study has a few limitations that should not be ignored. Firstly, as we used an online cross-sectional survey, there is a chance of recall bias. In addition, a self-reported questionnaire can always result in information bias. Moreover, despite the fact that the surveyed group of nursing students was representative of the population, there is a risk that the questionnaire was completed by a group of students supporting vaccination and those already vaccinated, while those opposing vaccination did not take part in the online survey, and COVID-19 vaccination may have resulted in a low anxiety level in the investigated group of students.

## 5. Conclusions

The studied group of Polish nursing students, after more than a year into the pandemic, showed low levels of anxiety, comparable to that in the group of medical students before the pandemic. The low levels of anxiety most probably resulted from being vaccinated against COVID-19 as quickly as possible, as well as having good knowledge about vaccination and access to up-to-date knowledge at one’s home university. In view of the research results obtained, vaccination against COVID-19 should be promoted among students and, if possible, the vaccination process should be facilitated in terms of organization and logistics. This may not only affect reaching herd immunity and minimizing the risk of the spread of infection, but it can also reduce the level of anxiety. Low levels of anxiety may improve the overall quality of life of students, as well as study efficiency, and may increase students’ readiness to enter the nursing profession after graduation.

## Figures and Tables

**Table 1:** Characteristics of study group (*N* = 790).

Nursing Department	
Pomeranian Medical University	238 (30.1)
Medical University of Warsaw	114 (14.4)
Medical University of Gdańsk	86 (10.9)
Jagiellonian University Medical College	65 (8.2)
Poznan University of Medical Sciences	61 (7.7)
Medical University of Lodz	46 (5.8)
Wroclaw Medical University	39 (4.9)
Medical University of Białystok	36 (4.6)
Medical University of Lublin	35 (4.4)
Medical University of Silesia	33 (4.2)
Jan Kochanowski University Medical College	20 (2.5)
University of Physical Education in Warsaw	17 (2.2)
Year of Study, *N* (%)	
1	316 (40.0)
2	331 (41.9)
3	143 (18.1)
Gender, *N* (%)	
Female	721 (91.3)
Male	57 (7.2)
Refusal to answer	12 (1.5)
Age (Years)	
M ± SD	22.4 ± 5.00
Range	19.0–53.0
Residence, *N* (%)	
Alone	101 (12.8)
With relatives/family/friends (excluding seniors)	550 (69.6)
With relatives/family/friends (including seniors)	139 (17.6)
Living with a person at higher risk of COVID-19, *N* (%)	
Yes	234 (29.6)
No	556 (70.4)
COVID-19 infection from people in the immediate environment, *N* (%)	
Yes, acute or very acute	179 (22.7)
Yes, but rather mild	390 (49.4)
No	171 (21.6)
I do not know	50 (6.3)
COVID-19 infection, *N* (%)	
Yes (acute symptoms of infection)	21 (2.7)
Yes (mild symptoms of infection)	71 (9.0)
Yes (no symptoms of infection)	11 (1.4)
Probably (no test confirmation)	174 (22.0)
No	333 (42.2)
I do not know	180 (22.8)

M—mean; SD—standard deviation.

**Table 2:** Anxiety level and willingness to get a COVID-19 vaccine.

	No Anxiety		Mild Anxiety		Moderate Anxiety		Severe Anxiety		*χ* ^2^	*p*-Value *
	*N*	%	*N*	%	*N*	%	*N*	%		
Vaccination against COVID-19										
No	76	23.7	56	23.7	33	20.6	13	18.3	8.807	0.185
Yes, mRNA	189	58.9	136	57.6	111	69.4	46	64.8		
Yes, vector-based vaccine	56	17.4	44	18.6	16	10.0	12	16.9		
Selected vaccination site										
Workplace	24	9.8	21	11.7	13	10.2	7	12.1	4.959	0.549
University	199	81.2	147	81.7	110	86.6	47	81.0		
Other	22	9.0	12	6.7	4	3.1	4	6.9		

* Chi-squared test.

**Table 3:** Anxiety level and access to current information on vaccination at university.

	No Anxiety		Mild Anxiety		Moderate Anxiety		Severe Anxiety		*χ* ^2^	*p*-Value *
	*N*	%	*N*	%	*N*	%	*N*	%		
Has the university provided access to up-to-date knowledge on vaccination?										
Yes	132	41.0	104	43.9	59	37.1	23	32.4	10.500	0.105
No	108	33.5	74	31.2	65	40.9	35	49.3		
I do not know	82	25.5	59	24.9	35	22.0	13	18.3		

* Chi-squared test.

**Table 4:** Frequency of use of sources of information on vaccination and anxiety levels.

	No Anxiety		Mild Anxiety		Moderate Anxiety		Severe Anxiety		*F*_(3, 785)_	*p*-Value *
	M	SD	M	SD	M	SD	M	SD		
(a) Websites of institutions and organizations related to health protection	3.33	1.95	3.36	1.85	3.45	1.80	3.63	1.98	0.595	0.618
(b) Non-health-related websites	1.79	1.69	2.06	1.79	2.05	1.77	2.04	1.79	1.519	0.208
(c) Specialized professional journals (online and in paper)	2.09	2.00	2.59	1.89	2.47	1.89	2.89	2.13	4.911	0.002
(d) Social media	2.49	1.94	2.91	1.98	2.87	1.92	2.86	2.02	2.675	0.046
(e) Blogs and vlogs of healthcare specialists	2.26	2.06	2.84	1.99	2.67	2.09	3.10	2.04	5.451	0.001
(f) Non-specialist blogs and vlogs	0.64	1.16	0.97	1.50	0.81	1.26	1.03	1.47	3.741	0.011
(g) Web portals (e.g., Onet and Wirtualna Polska)	1.48	1.67	1.67	1.71	1.78	1.82	1.59	1.65	1.289	0.277
(h) Radio/television	1.94	1.88	2.15	1.87	2.15	1.94	1.96	1.90	0.756	0.519
(i) Classes at university	3.05	1.81	3.13	1.84	3.16	2.01	2.77	1.83	0.823	0.481
(j) Workplace	1.50	2.13	1.70	2.12	1.88	2.32	1.80	2.32	1.212	0.304
(k) Other students at university	2.29	1.82	2.70	1.68	2.66	1.73	2.82	1.93	3.739	0.011
(l) Colleagues	1.35	2.00	1.53	1.96	1.66	2.06	1.52	2.14	0.918	0.432
(m) Family/friends not related to health protection	1.58	1.78	1.79	1.72	1.70	1.69	1.75	2.01	0.728	0.535

* One-way ANOVA.

**Table 5:** Knowledge about vaccination and anxiety levels.

Statement	No Anxiety		Mild Anxiety		Moderate Anxiety		Severe Anxiety		*χ* ^2^	*p*-Value *
	*N*	%	*N*	%	*N*	%	*N*	%		
After recovering from COVID-19 infection, vaccination is no longer necessary										
Incorrect	40	14.3	25	10.7	14	8.5	12	10.8	3.811	0.283
Correct	239	85.7	209	89.3	150	91.5	99	89.2		
mRNA vaccines contain viral particles.										
Incorrect	166	59.5	137	58.5	106	65.0	59	53.6	3.727	0.292
Correct	113	40.5	97	41.5	57	35.0	51	46.4		
You should avoid contact with people at higher risk of COVID-19 illness for 24 h after COVID-19 vaccination, as there is a possibility of infecting them with viral particles from the vaccine										
Incorrect	88	31.5	66	28.2	52	31.7	30	27.3	1.292	0.731
Correct	191	68.5	168	71.8	112	68.3	80	72.7		
A person vaccinated with two doses no longer needs to wear a face mask										
Incorrect	30	10.8	21	9.0	16	9.8	11	9.9	0.456	0.928
Correct	249	89.2	213	91.0	148	90.2	100	90.1		
It was proven that, as with other vaccinations, vaccine protection against SARS-CoV-2 is life-long										
Incorrect	53	19.0	47	20.1	38	23.2	24	21.6	1.211	0.750
Correct	226	81.0	187	79.9	126	76.8	87	78.4		
When vaccinating, vaccines from two different manufacturers can be used for the first- and second-dose administrations of the vaccine										
Incorrect	43	15.4	29	12.4	24	14.6	13	11.7	1.498	0.683
Correct	236	84.6	205	87.6	140	85.4	98	88.3		
Complete vaccination protection develops within 7–14 days after the second dose of the vaccine was administered										
Incorrect	91	32.6	69	29.5	50	30.5	41	36.9	2.141	0.544
Correct	188	67.4	165	70.5	114	69.5	70	63.1		
After reaching the threshold of herd immunity, COVID-19 will most likely become one of the seasonal diseases										
Incorrect	95	34.1	98	41.9	61	37.2	49	44.1	5.075	0.166
Correct	184	65.9	136	58.1	103	62.8	62	55.9		
Currently, only mRNA vaccines are marketed in the European Union										
Incorrect	77	27.6	63	27.0	49	29.9	28	25.2	0.776	0.855
Correct	202	72.4	170	73.0	115	70.1	83	74.8		
The pandemic will continue until more than 40% of the population has become immunized naturally (through disease) or artificially (through vaccination)										
Incorrect	195	69.9	161	69.1	115	70.1	74	67.3	0.315	0.957
Correct	84	30.1	72	30.9	49	29.9	36	32.7		

* Chi-squared test.

**Table 6:** Contraindications to vaccination and anxiety levels.

	No Anxiety		Mild Anxiety		Moderate Anxiety		Severe Anxiety		*χ* ^2^	*p*-Value *
	*N*	%	*N*	%	*N*	%	*N*	%		
Patients after cell, tissue, and organ transplants										
No	109	39.1	107	45.7	72	43.9	48	43.2	2.490	0.477
Yes	170	60.9	127	54.3	92	56.1	63	56.8		
Dialysis patients										
No	192	68.8	168	71.8	110	67.1	80	72.1	1.420	0.701
Yes	87	31.2	66	28.2	54	32.9	31	27.9		
Patients with neoplastic diseases										
No	149	53.4	109	46.6	85	51.8	66	59.5	5.446	0.142
Yes	130	46.6	125	53.4	79	48.2	45	40.5		
Pregnant women										
No	124	44.4	101	43.2	73	44.5	52	46.8	0.415	0.937
Yes	155	55.6	133	56.8	91	55.5	59	53.2		
Breastfeeding women										
No	161	57.7	122	52.1	91	55.5	68	61.3	3.010	0.390
Yes	118	42.3	112	47.9	73	44.5	43	38.7		
Patients requiring prolonged mechanical ventilation										
No	209	74.9	169	72.2	120	73.2	80	72.1	0.595	0.898
Yes	70	25.1	65	27.8	44	26.8	31	27.9		
Patients with hypersensitivity to vaccine components										
No	29	10.4	16	6.8	16	9.8	7	6.3	3.079	0.380
Yes	250	89.6	218	93.2	148	90.2	104	93.7		

* Chi-squared test.

## References

[1] (2020). The Social Impacts of COVID-19 Reset Not Restart: Taking Advantage of a Crisis for Social Change. https://www2.deloitte.com/content/dam/Deloitte/au/Documents/Economics/deloitte-au-dae-social-impact-of-covid-19-100820.pdf.

[2] Torales J., O’Higgins M., Castaldelli-Maia J.M., Ventriglio A. (2020). The outbreak of COVID-19 coronavirus and its impact on global mental health. Int. J. Soc. Psychiatry.

[3] Fortgang R.G., Wang S.B., Millner A.J., Reid-Russell A., Beukenhorst A.L., Kleiman E.M., Bentley K.H., Zuromski K.L., Al-Suwaidi M., Bird S.A. (2021). Increase in Suicidal Thinking during COVID-19. Clin. Psychol. Sci..

[4] Panchal N., Kamal R., Cox C., Garfield R. (2021). The Implications of COVID-19 for Mental Health and Substance Use. https://www.kff.org/coronavirus-covid-19/issue-brief/the-implications-of-covid-19-for-mental-health-and-substance-use/.

[5] Jungmann S.M., Witthöft M. (2020). Health anxiety, cyberchondria, and coping in the current COVID-19 pandemic: Which factors are related to coronavirus anxiety?. J. Anxiety Disord..

[6] Killgore W.D.S., Cloonan S.A., Taylor E.C., Fernandez F., Grandner M.A., Dailey N.S. (2020). Suicidal ideation during the COVID-19 pandemic: The role of insomnia. Psychiatry Res..

[7] Rodríguez-Hidalgo A.J., Pantaleón Y., Dios I., Falla D. (2020). Fear of COVID-19, Stress, and Anxiety in University Undergraduate Students: A Predictive Model for Depression. Front. Psychol..

[8] Mertens G., Gerritsen L., Duijndam S., Salemink E., Engelhard I.M. (2020). Fear of the coronavirus (COVID-19): Predictors in an online study conducted in March 2020. J. Anxiety Disord..

[9] Agu C.F., Stewart J., McFarlane-Stewart N., Rae T. (2021). COVID-19 pandemic effects on nursing education: Looking through the lens of a developing country. Int. Nurs. Rev..

[10] Dewart G., Corcoran L., Thirsk L., Petrovic K. (2020). Nursing education in a pandemic: Academic challenges in response to COVID-19. Nurse Educ. Today.

[11] Lovrić R., Farčić N., Mikšić Š., Včev A. (2020). Studying During the COVID-19 Pandemic: A Qualitative Inductive Content Analysis of Nursing Students’ Perceptions and Experiences. Educ. Sci..

[12] Singh H.K., Joshi A., Malepati R.N., Najeeb S., Balakrishna P., Pannerselvam N.K., Singh Y.K., Ganne P. (2021). A survey of E-learning methods in nursing and medical education during COVID-19 pandemic in India. Nurse Educ. Today.

[13] Fitzgerald A., Konrad S. (2021). Transition in learning during COVID-19: Student nurse anxiety, stress, and resource support. Nurs. Forum.

[14] García-González J., Ruqiong W., Alarcon-Rodriguez R., Requena-Mullor M., Ding C., Ventura-Miranda M.I. (2021). Analysis of Anxiety Levels of Nursing Students Because of e-Learning during the COVID-19 Pandemic. Healthcare.

[15] Li W., Gillies R., He M., Wu C., Liu S., Gong Z., Sun H. (2021). Barriers and facilitators to online medical and nursing education during the COVID-19 pandemic: Perspectives from international students from low- and middle-income countries and their teaching staff. Hum. Resour. Health.

[16] Kochuvilayil T., Fernandez R.S., Moxham L.J., Lord H., Alomari A., Hunt L., Middleton R., Halcomb E.J. (2021). COVID-19: Knowledge, anxiety, academic concerns and preventative behaviours among Australian and Indian undergraduate nursing students: A cross-sectional study. J. Clin. Nurs..

[17] Sun Y., Wang D., Han Z., Gao J., Zhu S., Zhang H. (2020). Disease Prevention Knowledge, Anxiety, and Professional Identity during COVID-19 Pandemic in Nursing Students in Zhengzhou, China. J. Korean Acad. Nurs..

[18] Dalcalı B.K., Durgun H., Taş A.S. (2021). Anxiety levels and sleep quality in nursing students during the COVID-19 pandemic. Perspect. Psychiatr. Care.

[19] Savitsky B., Findling Y., Ereli A., Hendel T. (2020). Anxiety and coping strategies among nursing students during the covid-19 pandemic. Nurse Educ. Pract..

[20] Uğurlu Y.K., Değirmenci D.M., Durgun H., Uğur H.G. (2021). The examination of the relationship between nursing students’ depression, anxiety and stress levels and restrictive, emotional, and external eating behaviors in COVID-19 social isolation process. Perspect. Psychiatr. Care.

[21] Salameh B., Basha S., Basha W., Abdallah J. (2021). Knowledge, Perceptions, and Prevention Practices among Palestinian University Students during the COVID-19 Pandemic: A Questionnaire-Based Survey. Inq. J. Health Care Organ. Provis. Financ..

[22] ElBagoury M., Tolba M.M., Nasser H.A., Jabbar A., Elagouz A.M., Aktham Y., Hutchinson A. (2021). The find of COVID-19 vaccine: Challenges and opportunities. J. Infect. Public Health.

[23] Wang X., Du Z., Johnson K.E., Pasco R.F., Fox S.J., Lachmann M., McLellan J.S., Meyers L.A. (2021). Effects of COVID-19 Vaccination Timing and Risk Prioritization on Mortality Rates, United States. Emerg. Infect. Dis..

[24] Leshem E., Wilder-Smith A. (2021). COVID-19 vaccine impact in Israel and a way out of the pandemic. Lancet.

[25] Haas E.J., Angulo F.J., McLaughlin J.M., Anis E., Singer S.R., Khan F., Brooks N., Smaja M., Mircus G., Pan K. (2021). Impact and effectiveness of mRNA BNT162b2 vaccine against SARS-CoV-2 infections and COVID-19 cases, hospitalisations, and deaths following a nationwide vaccination campaign in Israel: An observational study using national surveillance data. Lancet.

[26] Lukasik K.M., Waris O., Soveri A., Lehtonen M., Laine M. (2019). The Relationship of Anxiety and Stress With Working Memory Performance in a Large Non-depressed Sample. Front. Psychol..

[27] Wawrzuta D., Jaworski M., Gotlib J., Panczyk M. (2021). What Arguments against COVID-19 Vaccines Run on Facebook in Poland: Content Analysis of Comments. Vaccines.

[28] Spitzer R.L., Kroenke K., Williams J.B.W., Löwe B. (2006). A Brief Measure for Assessing Generalized Anxiety Disorder. Arch. Intern. Med..

[29] Löwe B., Decker O., Müller S., Brähler E., Schellberg D., Herzog W., Herzberg P.Y. (2008). Validation and standardization of the Generalized Anxiety Disorder Screener (GAD-7) in the general population. Med. Care.

[30] Swinson R.P. (2006). The GAD-7 scale was accurate for diagnosing generalised anxiety disorder. Evid. Based. Med..

[31] Zhong Q.-Y., Gelaye B., Zaslavsky A.M., Fann J.R., Rondon M.B., Sánchez S.E., Williams M.A. (2015). Diagnostic Validity of the Generalized Anxiety Disorder-7 (GAD-7) among Pregnant Women. PLoS ONE.

[32] Rutter L.A., Brown T.A. (2017). Psychometric Properties of the Generalized Anxiety Disorder Scale-7 (GAD-7) in Outpatients with Anxiety and Mood Disorders. J. Psychopathol. Behav. Assess..

[33] Bártolo A., Monteiro S., Pereira A. (2017). Factor structure and construct validity of the Generalized Anxiety Disorder 7-item (GAD-7) among Portuguese college students. Cad. Saude Publica.

[34] Hasson F., Keeney S., McKenna H. (2000). Research guidelines for the Delphi survey technique. J. Adv. Nurs..

[35] Geldsetzer P. (2020). Use of Rapid Online Surveys to Assess People’s Perceptions During Infectious Disease Outbreaks: A Cross-sectional Survey on COVID-19. J. Med. Internet Res..

[36] Hlatshwako T.G., Shah S.J., Kosana P., Adebayo E., Hendriks J., Larsson E.C., Hensel D.J., Erausquin J.T., Marks M., Michielsen K. (2021). Online health survey research during COVID-19. Lancet Digit. Health.

[37] Lun K.W., Chan C., Ip P.K., Ma S.Y., Tsai W., Wong C., Wong C.H., Wong T., Yan D. (2018). Depression and anxiety among university students in Hong Kong. Hong Kong Med. J..

[38] Farrer L.M., Gulliver A., Bennett K., Fassnacht D.B., Griffiths K.M. (2016). Demographic and psychosocial predictors of major depression and generalised anxiety disorder in Australian university students. BMC Psychiatry.

[39] Quek T.T.-C., Tam W.W.-S., Tran B.X., Zhang Z., Ho C.S. (2019). The Global Prevalence of Anxiety Among Medical Students: A Meta-Analysis. Int. J. Environ. Res. Public Health.

[40] Gotlib J., Sobierajski T., Jaworski M., Wawrzuta D., Borowiak E., Dobrowolska B., Dyk D., Gaworska-Krzemińska A., Grochans E., Kózka M. (2021). “Vaccinate, Do Not Hesitate!”. Vaccination Readiness against COVID-19 among Polish Nursing Undergraduate Students: A National Cross-Sectional Survey. Vaccines.

